# Pediatric moderate and severe traumatic brain injury – A national, population-based cohort study

**DOI:** 10.1007/s00068-025-02960-1

**Published:** 2025-10-30

**Authors:** Trym Kolstø, David Werner, Kenneth Thorsen, Clemens Weber

**Affiliations:** 1 Department of Neurology, Stavanger University Hospital, Stavanger, Norway; 2 Department of Neurosurgery, Stavanger University Hospital, Stavanger, Norway; 3 Department of Gastrointestinal Surgery, Stavanger University Hospital, Stavanger, Norway; 4 Section for Traumatology, Surgical clinic, Stavanger University Hospital, Stavanger, Norway

**Keywords:** Pediatric traumatic brain injury, Moderate TBI, Severe TBI, Abbreviated injury scale, Glasgow outcome score, Functional outcome

## Abstract

**Purpose:**

This study aims to investigate the epidemiological characteristics, injury mechanisms, and clinical outcomes of children with moderate and severe traumatic brain injury (TBI), using population-based data from a national trauma registry.

**Methods:**

This nationwide observational cohort study includes all pediatric patients (0–17 years) with moderate and severe TBI registered in the Norwegian Trauma Registry between January 1, 2017, and December 31, 2020.

**Results:**

A total of 348 pediatric patients with moderate (*n* = 289, 83%) and severe *n* = 59, 17%) TBI were analyzed. The most common injury mechanism were high-energy falls (33%) and road traffic accidents (29%). The 30-day mortality rate was 3%, significantly higher in severe TBI (12%) than moderate TBI (1%, *p* < 0,001). Patients with severe TBI had longer hospital stays and poorer functional recovery than those with moderate TBI.

**Conclusion:**

This population-based study on pediatric TBI patients over a period of 4 years revealed a relatively low mortality rate. In 4 out of 5 patients moderate or severe TBI resulted in a good outcome with moderate, low or no disability. Advancing the understanding of evidence-based interventions aiming at preventing both primary and secondary injuries associated with pediatric TBI is essential for optimizing recovery and promoting functional independence in these young patients.

## Introduction

Traumatic brain injury (TBI) is a major cause of disability and death among pediatric trauma patients [1, 2]. Pediatric TBI is a significant health issue with the potential to cause profound and long-lasting cognitive, physical and psychosocial impairments [3]. It is estimated that over 50,000 children are hospitalized for TBI each year in the USA [4, 5] TBI also leads to numerous outpatient visits, emergency department visits and other healthcare encounters [6]. According to the Center for disease Control and Prevention (CDC), more than 5 million people are living with a TBI related disability in the USA today [7]. Looking at European statistics, it becomes evident that in 2012, approximately 82,000 deaths resulted from traumatic brain injuries [8].

However, despite these results, ongoing efforts with quick interventions and effective treatments lead to more than half of children with moderate and severe TBI to have positive long-term outcomes in the pediatric population [9].

National trauma registries are valuable data sources with prospectively collected data. The National Trauma Registry of Norway (NTR) contains information on all potentially seriously injured patients treated by all public hospitals delivering trauma care in Norway, including prehospital services. The registry is designed to monitor and improve the quality and outcome of trauma care delivered by Norwegian hospitals and their trauma services and has shown excellent data agreement [10]. This study aims to investigate the epidemiological characteristics and clinical outcomes of children with moderate and severe traumatic brain injury (TBI) using population-based data from a national trauma registry.

## Materials and methods

### Ethics

This research project was approved by the Regional Committee for Medical and Health Research Ethics of Western Norway (REC-ID 143902), and the scientific council of the NTR The Strengthening the Reporting of Observational studies in the Epidemiology (STROBE) guidelines were adhered to [11].

### Study design and period

The research design is a nationwide, observational cohort study based on prospectively collected data from the national trauma registry. The study period includes all patients under 18 years of age, with severe TBI included in the NTR, between January 1, 2017, and December 31, 2020.

### Data source

The data for this study were retrieved from the NTR, which is a comprehensive national database aimed at monitoring the quality of trauma treatment and to facilitating continuous improvement of the trauma care chain in Norway. The registry covers the entire Norwegian population within a nationwide, publicly funded, single-player healthcare system. All Norwegian hospitals (*n* = 38) admitting trauma patients with severe injury or suspected severe injury routinely deliver data to the trauma registry, ensuring nationwide individual coverage of over 95% and approximately 98% data completeness of key variables [12]. The core dataset of the trauma registry is based on the Utstein template for uniform reporting of data following major trauma [13].

### Study population

**Fig. 1:**
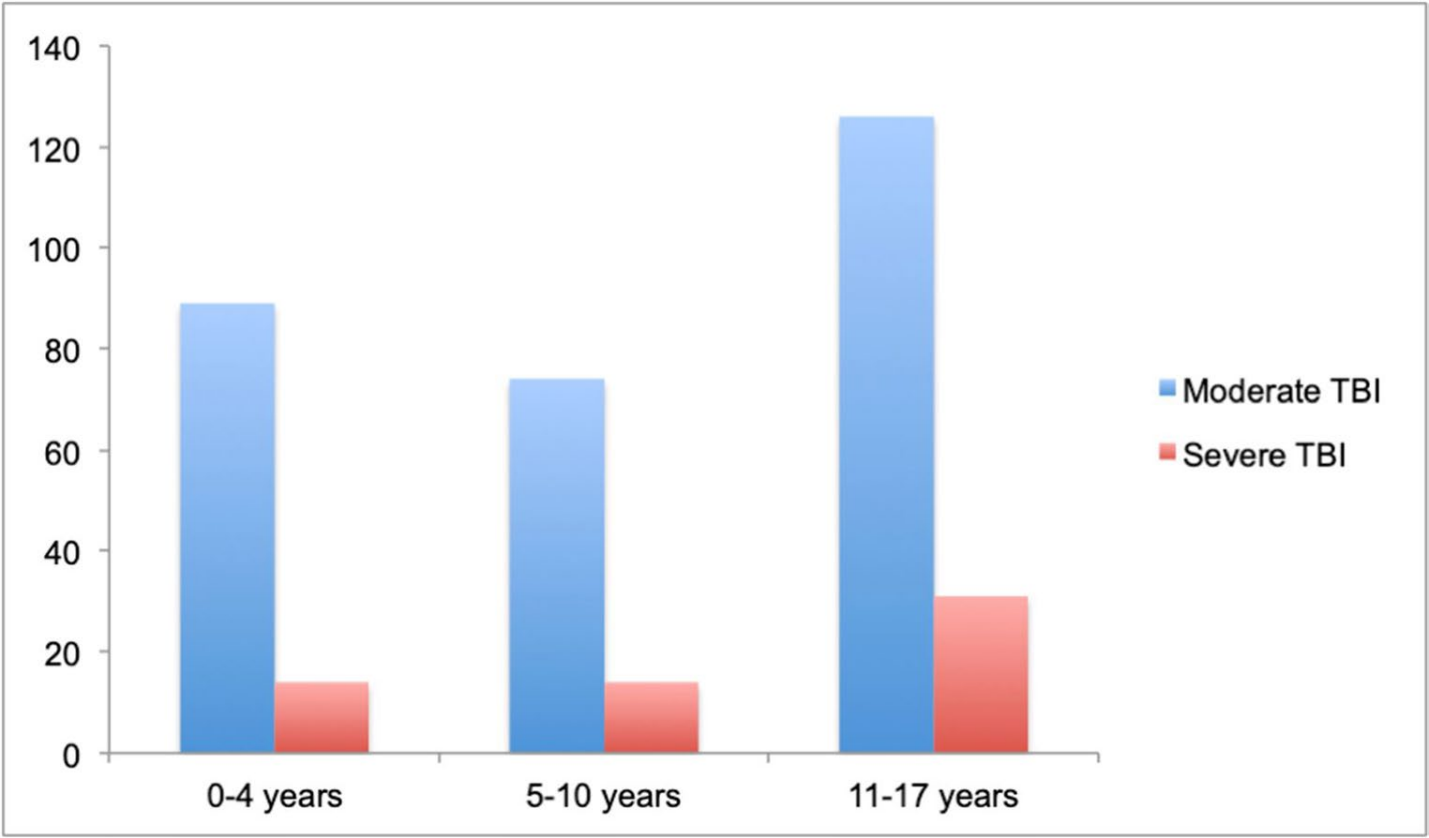
Number of patients with moderate and severe TBI according to age groups

All pediatric patients (aged 0–17 years) during the 4-years observation period were identified from the NTR. In 2023, a total of 34 emergency hospitals with trauma functions, all part of the four regional trauma centers, reported data to the NTR, achieving a coverage rate of 92% [14]. In this analysis, TBI severity was classified according to the maximal Abbreviated Injury score (AIS) for head injury into moderate TBI (AIS 3–4) and severe TBI (AIS ≥ 5) [15],. Traditionally, head injury is classified by the Head Injury Severity Scale into minimal, mild, moderate and severe TBI [16]. However, classifying TBI according to Glasgow Coma Scale (GCS) carries the risk of including patients with reduced GCS according to other causes than TBI (such as intoxication, other injuries, medical and iatrogenic causes). Classifying TBI by AIS is based on actual anatomical injuries, which makes it more reliable for epidemiological Fig.1 research and was therefore chosen for this study.

### Outcome measures

The outcome measures of this study were 30-day mortality rates, in-house mortality rates and discharge destination after acute care. The 30-day mortality rate was defined as mortality rate at 30 days after injury as registered in the registry. In-hospital mortality rate was defined as mortality rate at the definite care hospital. Discharge destination was registered after discharge from acute treatment at the definite care hospital, including:

- Home.
- Rehabilitation institution.
- Other intensive care ward.
- Other hospital ward.
- Other.

All statistical analyses were performed with SPSS version 28.0 (IBM, USA). The medians, percentages, and interquartile range (IQR) were used to describe the entire patient cohort, as well as the moderate and severe TBI cohorts. The chi-square test was used for categorical variables, and the independent samples median test was used for continuous variables. Statistical significance was defined as *p* < 0.050.

## Results

During the study period a total of 348 pediatric patients with moderate and severe TBI were registered in the NTR. Of these, 289 (83%) were classified as moderate TBI and 59 (17%) classified as severe TBI. Patient demographics and injury data for both patient cohorts are presented in Table 1.

**Table 1:** Patient and injury characteristics of pediatric traumatic brain injury

Variables	All patients	Moderate TBI	Severe TBI	*p*-value
No. of patients (%)	348	289 (83%)	59 (17%)	
Age in years, median (IQR)	9 (4–15)	8 (4–14,5)	12 (5–15)	0,126
Gender, No. (%)				
Male	215 (62%)	175 (61%)	40 (68%)	0,297
Female	133 (38%)	114 (39%)	19 (32%)	
Dominating type of injury, No. (%)				
Blunt	336 (100%)	284 (100%)	52 (100%)	0,669
Penetrating	1 (0%)	1 (0%)	0	
Mechanism of injury, No. (%)				
RTA	101 (29%)	77 (27%)	24 (41%)	*0*,*031*
Hit by blunt object	54 (16%)	47 (16%)	7 (12%)	0,396
Low energy fall	57 (16%)	51 (18%)	6 (10%)	0,157
High energy fall	116 (33%)	101 (35%)	15 (25%)	0,157
Other	20 (6%)	13 (5%)	7 (12%)	*0*,*026*
Intention of injury, No. (%)				
Unintentional	330 (98%)	278 (98%)	52 (100%)	0,521
Self-inflicted	5 (2%)	5 (2%)	0	
GCS at scene, median (IQR)	14,0 (9,0–15,0)	14,0 (12,0–15,0)	6,0 (3,0–12,75)	*< 0*,*001*

*IQR*: Interquartile range;* RTA* – Road traffic accidents;* GCS* – Glasgow Coma Scale; *ED* - Emergency Department

The most common mechanism of injury among all the patients was high energy falls (33%) followed by road traffic accidents (29%). In the moderate TBI group, road traffic accidents accounted for 27% of injuries, versus 41% in the severe TBI group (*p* < 0.031).

Patients with severe TBI had significantly higher median ISS (30 vs. 11; *p* < 0.001) and lower median GCS score than patients with moderate TBI (6.0 vs. 14.0; *p* < 0.001). Further injury severity and length of treatment data are presented in Table 2.

**Table 2:** Comparison of injury severity, Glasgow coma scale and length of hospital stay (LoS) in moderate vs. Severe TBI

Variables	All patients	Moderate TBI	Severe TBI	*p*-value
ISS, median (IQR)	14 (10–22)	11 (10–17)	30 (26–38)	*< 0*,*001*
TBI severity GCS, No. (%)				
Mild	211 (67%)	194 (71%)	17 (38%)	*< 0*,*001*
Moderate	59 (19%)	49 (18%)	10 (18%)	0,493
Severe	47 (15%)	29 (11%)	18 (40%)	*< 0*,*001*
LoS, median (IQR)	3,0 (1,0–5,0)	2,0 (1,0–5,0)	5,0 (1,0–13,0)	*0*,*032*

*ISS* – Injury Severity Score

The 30-day mortality rate for the whole patient cohort was 3% and significantly lower in the moderate TBI cohort compared to the severe TBI cohort (1% vs. 12%, *p* < 0.001). Regarding functional outcomes, presented in Table 3, the severe disability category encompassed a total of 54 patients (16%). Among these, 29 (10%) were from the moderate TBI group, and 25 (42%) belonged to the severe TBI group (*p* < 0.001). Moderate disability was the most prevalent category, encompassing a total of 220 patients (63%), with 197 (68%) from the moderate TBI group and 23 (39%) from the severe TBI group (*p* < 0.001). The low or no disability category included a total of 62 patients (18%), with 58 (20%) from the moderate TBI group and a smaller proportion of 4 (7%) from the severe TBI group (*p* = 0.014). In terms of discharge destinations, most patients were discharged home, totaling 217 (62%). This included 195 (68%) from the moderate TBI category and 22 (37%) from the severe TBI category (*p* < 0.001). Furthermore, 31 (9%) patients were discharged to rehabilitation centers, with 14 (5%) originating from the moderate TBI group and 17 (29%) from the severe TBI group (*p* < 0.001).

**Table 3:** Outcome variables of severe traumatic brain injury

Variables	All patients	Moderate TBI	Severe TBI	*p*-value
30-day mortality, No. (%)	11 (3%)	4 (1%)	7 (12%)	*< 0*,*001*
Glasgow Outcome Scale*, No. (%)				
Vegetative state	3 (1%)	2 (1%)	1 (2%)	0,999
Severe disability	54 (16%)	29 (10%)	25 (42%)	*< 0*,*001*
Moderate disability	220 (63%)	197 (68%)	23 (39%)	*< 0*,*001*
Low or no disability	62 (18%)	58 (20%)	4 (7%)	*0*,*014*
Discharge destination, No. (%)				
Home	217 (62%)	195 (68%)	22 (37%)	*< 0*,*001*
Rehabilitation	31 (9%)	14 (5%)	17 (29%)	*< 0*,*001*
Other ICU	68 (20%)	58 (20%)	10 (17%)	0,572
Other hospital ward	23 (7%)	20 (7%)	3 (5%)	0,528

*ICU* – Intensive Care Unit

* At time of discharge

## Discussion

This population-based study on pediatric TBI patients over a period of 4 years revealed a relatively low mortality rate of 3% (1% in moderate and 12% in severe TBI), with 17% of the patients having a poor outcome resulting in vegetative state or severe disability. However, in 4 out of 5 patients, moderate or severe TBI resulted in moderate, low, or no disability. As expected, pediatric patients with moderate TBI in this patient cohort fared better, with mainly favorable functional outcome. Patients in the severe TBI group showed a more profound neurological impairment and a more critical clinical presentation compared to those in the moderate TBI group. Additionally, patients with severe TBI had a longer length of stay (LoS) compared to those with moderate TBI.

Our data shows that there is a noticeable rise in the occurrence of both moderate and severe TBIs among individuals aged 11 to 17 years, compared to those below 11 years of age. These findings are consistent with the results observed in the population-based study conducted in Trondheim [17].

Lastly, the individual discharge destination findings underscore the distinct post-hospitalization care needs of patients with varying TBI severities, emphasizing the importance of tailored rehabilitation strategies.

In a parallel study investigating the epidemiology of pediatric moderate and severe TBI in the Netherlands, a significant similarity is apparent. Both study cohorts exhibited similar injury mechanisms, with falls and RTA being the predominant causes of TBI in both cases [18].

A notable disparity in recovery potential between moderate and severe TBI cases is evident in the population-based global outcome study conducted in Trondheim, Norway [17]. The data suggest that moderate TBI patients have a recovery potential of nearly 90%, while dropping to under 50% for severe TBI cases. However, as mentioned, assessing functional outcomes requires extended follow-up periods, indicating the need for further in-depth investigation in this domain. Unsurprisingly, the study also found that falls, and RTA were the most common injury mechanisms. Notably, their findings demonstrated that measuring the Glasgow Outcome Scale-Extended (GOS-E) in RTA cases resulted in comparatively poorer outcomes.

In a cohort study from our research group based on data from a regional trauma registry in Stavanger, Norway, the overall mortality rate was18%. In Trondheim, the calculated mortality rate among children in a similar age group was approximately 4.3% [17, 19]. When comparing our results, we observe a discrepancy in the mortality rate. This difference is likely due to our stringent inclusion criteria, which use AIS as a measure of TBI rather than GCS. Additionally, a more focused examination of the mortality rate among moderate TBI cases reveals a noteworthy distinction. The previous study conducted in Stavanger reported zero mortality among moderate TBI patients. In contrast, our study observed a total of 4 patients making the mortality rate 1%. This implies that the mortality rate in the previous study was primarily influenced by severe TBI cases, accounting for 38%, whereas our findings show a 12% mortality rate for this group.

The patient population in these studies differs slightly. We emphasized using a strict inclusion criterion based on AIS, head injury severity. The decision to use AIS is supported by the findings of Savitsky et al. [15], who demonstrated that an AIS score of 5 or higher offers superior predictive value of mortality outcomes. Their study also highlighted key limitations of relying on GCS alone, as various external factors, such as drug use, electrolyte imbalances, or non-neurological conditions (e.g., cardiac dysfunction), can influence GCS scores independently of any actual brain injury. Importantly the study also revealed a discrepancy between GCS and AIS classifications where only 35% of patients with an AIS of 5 or higher also had a GCS score of 3–8. However, the highest mortality rates were observed in patients for whom both AIS and GCS classified the injury as severe. This finding suggests that the combined use of both scoring systems may provide a more accurate prediction of mortality than relying on either tool alone. An important consideration for future clinical and epidemiological applications.

The evidence presented in this study underscores the importance of accurately assessing TBI severity for appropriate management and resource allocation. Early intervention and individualized rehabilitation programs are important consideration when aiming to improve outcomes [20]. Moreover, the findings emphasize the need for continued efforts in preventing high energy falls and RTA, among children. Implementing strategies such as child safety measures, educational programs, and environmental modifications can play a vital role in reducing the incidence and severity of falls and RTA, potentially reducing the burden of pediatric TBI.

### Strength and limitations

The reliability of this study is significantly bolstered by several key factors. Firstly, the fact that this study is conducted on a national level lends substantial credibility to their findings.

Secondly, the study uses data from an established and reliable source, namely the NTR. Relying on a well-recognized and thoroughly maintained database ensures the accuracy and consistency of the data collected, reinforcing the integrity of the study’s conclusions.

Thirdly, the strict inclusion criteria of a specific patient demographic, such as age and injury mechanism, coupled with a thorough categorization of TBI injury severity into distinct levels, offer a more inclusive sample of children with moderate and severe TBI, providing a broader representation of this population. We employed the Abbreviated Injury Scale (AIS) to delineate the degree of brain injury. This approach is more precise compared to using the GCS, as it allows us to exclude individuals influenced by drugs or other types of injuries, resulting in a more precisely defined group for our study. The prospective collection of data could minimize any potential biases. Overall, this study may provide valuable insights into the clinical characteristics and outcome of children with moderate and severe TBI, which could inform the development of improved prevention and treatment.

Limitations of this study includes a small sample size, the socio-economic factors, influencing the patient cohort, which should be considered when generalizing the findings. Furthermore, long-term functional data – including physical and psychological metrics, as well as patient-reported outcomes (PROMs) – are not available from the NTR and therefore could not be included in this study.

## Conclusion

In this population-based study, we observed that high-energy falls and RTA were among the most common causes of moderate to severe TBI in children aged 0–17 years.

When evaluating functional outcomes, it becomes evident that there is substantial potential for recovery, particularly in cases of moderate TBI. However, it’s important to note that most children experiencing moderate to severe TBI still face some sort of disability. Therefore, advancing our understanding of evidence-based intervention aimed at preventing both primary and secondary injuries associated with pediatric TBI is essential for optimizing neurocognitive recovery and promoting functional independence in these young patients.

## Data Availability

No datasets were generated or analysed during the current study.

## References

[1] Soreide K, Kruger AJ, Ellingsen CL, Tjosevik KE. Pediatric trauma deaths are predominated by severe head injuries during spring and summer. Scand J Trauma Resusc Emerg Med. 2009;17: 3. 19161621 10.1186/1757-7241-17-3 PMC2637226

[2] Andriessen TM, Horn J, Franschman G, van der Naalt J, Haitsma I, Jacobs B, et al. Epidemiology, severity classification, and outcome of moderate and severe traumatic brain injury: a prospective multicenter study. J Neurotrauma. 2011;28(10):2019–31. 21787177 10.1089/neu.2011.2034

[3] Popernack ML, Gray N, Reuter-Rice K. Moderate-to-severe traumatic brain injury in children: complications and rehabilitation strategies. J Pediatr Health Care. 2015;29(3):e1-7. 25449002 10.1016/j.pedhc.2014.09.003 PMC4409446

[4] Schneier AJ, Shields BJ, Hostetler SG, Xiang H, Smith GA. Incidence of pediatric traumatic brain injury and associated hospital resource utilization in the United States. Pediatrics. 2006;118(2):483–92. 16882799 10.1542/peds.2005-2588

[5] Dewan MC, Mummareddy N, Wellons JC 3rd, Bonfield CM. Epidemiology of global pediatric traumatic brain injury: qualitative review. World Neurosurg. 2016;91:497–509. e1. 27018009 10.1016/j.wneu.2016.03.045

[6] Chen C, Peng J, Sribnick EA, Zhu M, Xiang H. Trend of age-adjusted rates of pediatric traumatic brain injury in U.S. emergency departments from 2006 to 2013. Int J Environ Res Public Health. 2018. 10.3390/ijerph15061171. 29874782 10.3390/ijerph15061171 PMC6024977

[7] Flanagan SR. Invited commentary on centers for disease control and prevention report to congress: traumatic brain injury in the united states: epidemiology and rehabilitation. Arch Phys Med Rehabil. 2015;96(10):1753–5. 26184889 10.1016/j.apmr.2015.07.001

[8] Majdan M, Plancikova D, Brazinova A, Rusnak M, Nieboer D, Feigin V, et al. Epidemiology of traumatic brain injuries in europe: a cross-sectional analysis. Lancet Public Health. 2016;1(2):e76–83. 29253420 10.1016/S2468-2667(16)30017-2

[9] Shaklai S, Peretz R, Spasser R, Simantov M, Groswasser Z. Long-term functional outcome after moderate-to-severe paediatric traumatic brain injury. Brain Inj. 2014;28(7):915–21. 24826955 10.3109/02699052.2013.862739

[10] Naberezhneva N, Uleberg O, Dahlhaug M, Giil-Jensen V, Ringdal KG, Roise O. Excellent agreement of Norwegian trauma registry data compared to corresponding data in electronic patient records. Scand J Trauma Resusc Emerg Med. 2023;31(1):50. 37752614 10.1186/s13049-023-01118-5 PMC10521548

[11] von Elm E, Altman DG, Egger M, Pocock SJ, Gotzsche PC, Vandenbroucke JP, et al. The strengthening the reporting of observational studies in epidemiology (STROBE) statement: guidelines for reporting observational studies. J Clin Epidemiol. 2008;61(4):344–9. 18313558 10.1016/j.jclinepi.2007.11.008

[12] Dahlhaug M, Røise O. The annual report of the National Trauma Registry of Norway, Oslo University Hospital, Oslo, Norway. 2021.

[13] Ringdal KG, Coats TJ, Lefering R, Di Bartolomeo S, Steen PA, Roise O, et al. The Utstein template for uniform reporting of data following major trauma: a joint revision by SCANTEM, TARN, DGU-TR and RITG. Scand J Trauma Resusc Emerg Med. 2008;16: 7. 18957069 10.1186/1757-7241-16-7 PMC2568949

[14] Nordset VI-M, Holm KT, Azulay N. Nasjonalt traumeregister årsrapport for 2023.

[15] Savitsky B, Givon A, Rozenfeld M, Radomislensky I, Peleg K. Traumatic brain injury: it is all about definition. Brain Inj. 2016;30(10):1194–200. 27466967 10.1080/02699052.2016.1187290

[16] Stein SC, Spettell C. The head injury severity scale (HISS): a practical classification of closed-head injury. Brain Inj. 1995;9(5):437–44. 7550215 10.3109/02699059509008203

[17] Olsen M, Vik A, Lien E, Schirmer-Mikalsen K, Fredriksli O, Follestad T, et al. A population-based study of global outcome after moderate to severe traumatic brain injury in children and adolescents. J Neurosurg Pediatr. 2022;29(4):397–406. 35061977 10.3171/2021.11.PEDS21285

[18] Jochems D, van Rein E, Niemeijer M, van Heijl M, van Es MA, Nijboer T, et al. Epidemiology of paediatric moderate and severe traumatic brain injury in the Netherlands. Eur J Paediatr Neurol. 2021;35:123–9. 34687976 10.1016/j.ejpn.2021.10.004

[19] Weber c. Characteristics, image findings and clinical outcome of moderate and severe traumatic brain injury among severely injured children: a population-based cohort study. Eur J Trauma Emerg Surg. 2022. 10.1007/s00068-021-01820-y. 34999903 10.1007/s00068-021-01820-y

[20] Cope DN, Hall K. Head injury rehabilitation: benefit of early intervention. Arch Phys Med Rehabil. 1982;63(9):433–7. 7115044

